# Melanoma Cells Can Adopt the Phenotype of Stromal Fibroblasts and Macrophages by Spontaneous Cell Fusion *in Vitro*

**DOI:** 10.3390/ijms17060826

**Published:** 2016-06-02

**Authors:** Lajos V. Kemény, Zsuzsanna Kurgyis, Tünde Buknicz, Gergely Groma, Ádám Jakab, Kurt Zänker, Thomas Dittmar, Lajos Kemény, István B. Németh

**Affiliations:** 1 Department of Dermatology and Allergology, University of Szeged, Szeged 6720, Hungary; kemeny.lajos@gmail.com (L.V.K.); kurgyis.zsuzsanna@med.u-szeged.hu (Z.K.); buknicz.tunde@med.u-szeged.hu (T.B.); yakoo@freemail.hu (A.J.); 2 MTA-SZTE Dermatological Research Group, Szeged 6720, Hungary; groma.gergo@gmail.com; 3 Institute of Immunology & Experimental Oncology, Witten/Herdecke University, Witten 58453, Germany; kurt.zaenker@uni-wh.de (K.Z.); thomas.dittmar@uni-wh.de (T.D.)

**Keywords:** cell fusion, spontaneous melanoma, macrophage, fibroblast

## Abstract

After the removal of primary cutaneous melanoma some patients develop local recurrences, even after having histologically tumor-free re-excision. A potential explanation behind this phenomenon is that tumor cells switch their phenotype, making their recognition via standard histopathological assessments extremely difficult. Tumor-stromal cell fusion has been proposed as a potential mechanism for tumor cells to acquire mesenchymal traits; therefore, we hypothesized that melanoma cells could acquire fibroblast- and macrophage-like phenotypes via cell fusion. We show that melanoma cells spontaneously fuse with human dermal fibroblasts and human peripheral blood monocytes *in vitro.* The hybrid cells’ nuclei contain chromosomes from both parental cells and are indistinguishable from the parental fibroblasts or macrophages based on their morphology and immunophenotype, as they could lose the melanoma specific MART1 marker, but express the fibroblast marker smooth muscle actin or the macrophage marker CD68. Our results suggest that, by spontaneous cell fusion *in vitro*, tumor cells can adopt the morphology and immunophenotype of stromal cells while still carrying oncogenic, tumor-derived genetic information. Therefore, melanoma–stromal cell fusion might play a role in missing tumor cells by routine histopathological assessments.

## 1. Introduction

An increasing body of evidence indicates that cell fusion has not only a physiological role during tissue development and repair, but might also be a hidden force promoting tumor progression [1,2,3,4,5]. Fibroblasts, mesenchymal stem cells, and macrophages have been proposed as fusion partners of tumor cells [6,7,8]. Several studies have demonstrated that tumor-stromal hybrid cells can display more malignant properties than parental tumor cells [9,10], and the potential contribution of cell fusion to tumor heterogeneity has been suggested [11,12,13,14].

Most cell fusion studies are based on experiments on hybrid cells that originate from a single hybrid clone generated with electro- or chemical fusion followed by antibiotic selection. There are only a few studies focusing on spontaneous cell–cell fusion, which is a well-programmed event that requires a specific controlling system [15]. By artificially generating hybrid cells, there is the possibility that unique and important characteristics of hybrid cells might not be discovered. Furthermore, precise analysis of hybrid cell morphology and phenotype might also differ in a spontaneous and programmed event.

Terada *et al.* showed that bone-marrow-derived stem cells can adopt the phenotype of other cells by cell fusion [16]. Therefore, we hypothesized that tumor cells could adopt a stromal phenotype by fusing with peritumoral fibroblasts and macrophages and thus could become indistinguishable from tumor-associated fibroblasts and macrophages in the neighboring stroma. The detection of these “stealthy” tumor-stromal cell hybrids could have important diagnostic and therapeutic significance. For this reason, our aim was to establish a cell fusion model that is suitable for the characterization of spontaneously generated hybrid cells in tumorous diseases.

## 2. Results

### 2.1. Melanoma Cells Spontaneously Fuse with Fibroblasts and Monocytes in Vitro

**Figure 1:**
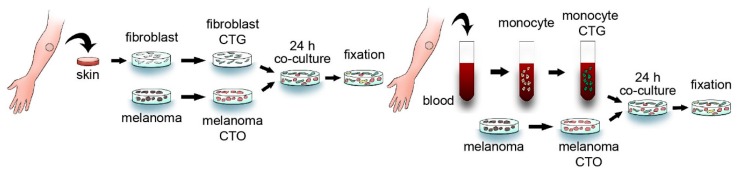
*In vitro* cell fusion model. Primary human dermal fibroblasts (**left panel**) or human peripheral blood-derived monocytes (**right panel**) labeled with CellTracker Green (CTG) were co-cultured with human melanoma cells labeled with CellTracker Orange (CTO) for 24 h. The samples were subsequently fixed and analyzed with a fluorescent microscope.

**Figure 2:**
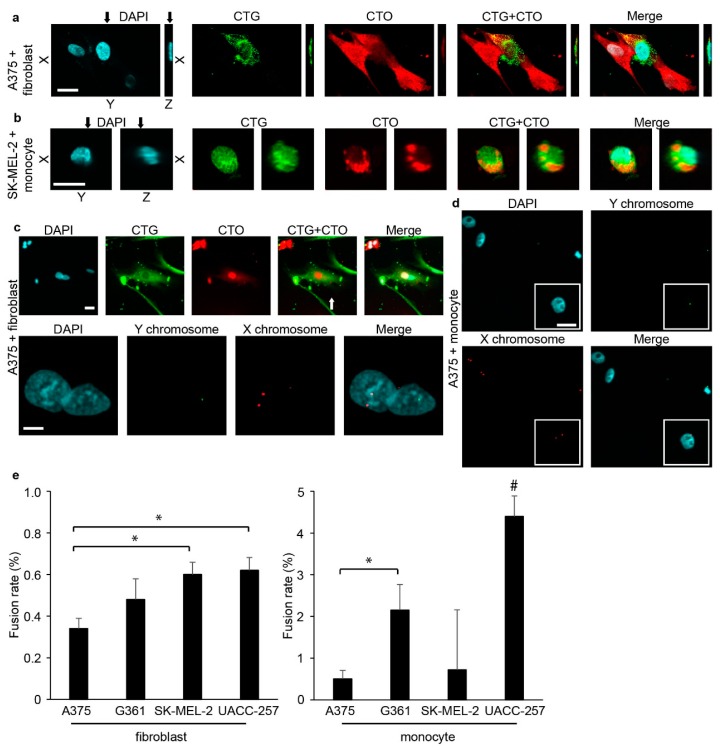
Melanoma cells spontaneously fused with primary human dermal fibroblasts and monocytes. (**a**,**b**) A melanoma (CTO)–fibroblast (CTG) (**a**) and a melanoma (CTO)–monocyte (CTG) (**b**) hybrid visualized with a confocal microscope in *X*-*Y* and *X*-*Z* planes. The *Z*-stacking confirms that double positivity does not result from cells lying on each other. Black arrows indicate the crossline of planes; white bars indicate 20 µm (**a**) and 10 µm (**b**). (**c**) A melanoma (CTO)–fibroblast (CTG) hybrid confirmed with confocal microscopy contains a nucleus from a female melanoma cell and another from a fibroblast from a male donor (**upper row**). Fluorescent *in situ* hybridization visualizing three X (red) and one Y (green) chromosome in the same cell (**lower row**). White bars indicate 30 µm (**upper row**) and 10 µm (**lower row**). (**d**) Fluorescent *in situ* hybridization visualizing two X (red) and one Y (green) chromosome in a melanoma (female)–monocyte (male) hybrid cell. White bar indicates 10 µm. (**e**) The percentage of hybrid cells compared to the total number of melanoma cells and fibroblasts or to the total number of melanoma cells and monocytes after 24 h of co-culture measured with flow cytometry. All investigated melanoma cell lines fuse spontaneously with human dermal fibroblasts (**left panel**) and peripheral blood-derived monocytes (**right panel**). Means of three experiments + SD are shown. * indicates significant differences between the marked groups. # indicates a significant difference between the marked group and all the other groups. *p* < 0.05. DAPI: 4′,6-Diamidin-2-phenylindol.

In order to investigate tumor–stromal cell fusion, we co-cultured various human melanoma cell lines either with primary human dermal fibroblasts or with freshly isolated human monocytes for 24 h. CellTracker Orange (CTO) and CellTracker Green (CTG) fluorescent vital dyes were used to identify melanoma cells and fibroblasts or monocytes, respectively (Figure 1). After 24 h, we observed the spontaneous formation of hybrid cells containing both CTO and CTG with laser scanning confocal microscopy. The subsequent three-dimensional reconstruction of such double positive cells demonstrated that the double fluorescence was detected in a single cell and not from two overlapping cells (Figure 2a,b and Supplementary Videos 1 and 2). To further confirm the fusion of melanoma cells and stromal cells at the genetic level, we used parental cells from donors of different genders and performed fluorescent *in situ* hybridization targeting the X and Y chromosomes in the hybrid cells to visualize and distinguish genetic material from both parental cells. For these experiments, A375 female melanoma cells were co-cultured with fibroblasts or monocytes from a male donor. A representative hybrid cell from each co-culture containing the sex chromosomes from both parental cells is displayed in Figure 2c,d. As also shown in Figure 2c, fluorescent *in situ* hybridization was performed on cell that has been previously identified as a hybrid cells containing both CTO and CTG, suggesting that both methods can identify the same hybrid cell. Fluorescent, four-dimensional, live-cell imaging also confirmed spontaneous cell fusion between a monocyte and a melanoma cell (Supplementary Video 3). The proportion of hybrid cells in the different melanoma cell lines was measured using flow cytometry and varied between 0.17%–0.31% and 0.5%–4.39% for fibroblasts and monocytes, respectively (Figure 2e and Supplementary Figures S1 and S2).

### 2.2. Hybrid Cells Adopt the Morphology and Phenotype of Stromal Cells

**Figure 3:**
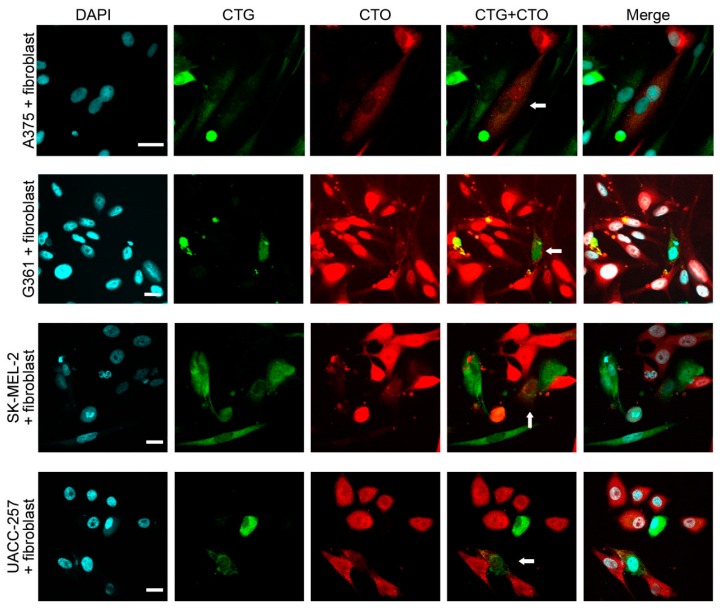
Melanoma–fibroblast hybrids are indistinguishable from stromal cells based on cell morphology. Spontaneously formed melanoma (CTO)–fibroblast (CTG) hybrids (white arrows) from different melanoma cell lines adopt the morphology of fibroblasts. White bars indicate 20 µm.

**Figure 4:**
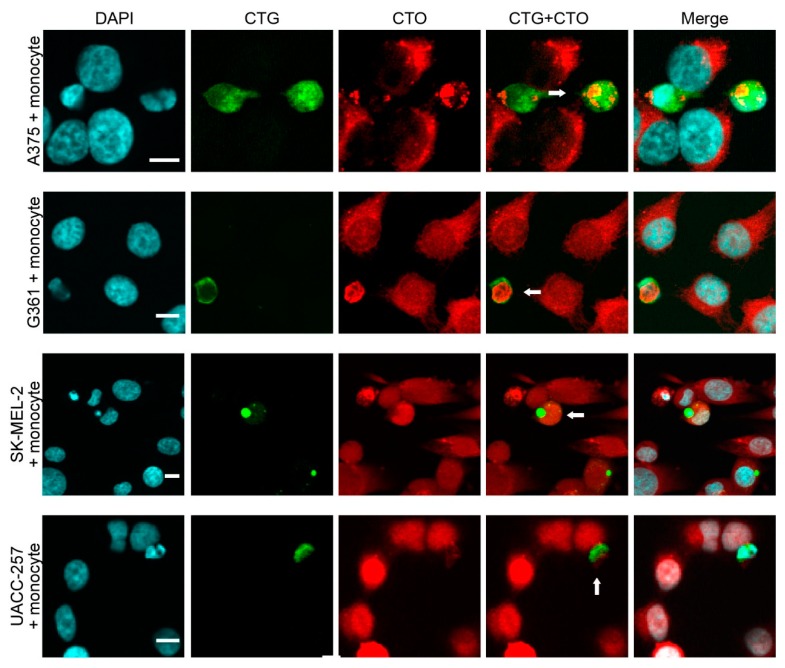
Melanoma–fibroblast and melanoma–monocyte hybrids are indistinguishable from stromal cells based on cell morphology. Spontaneously formed melanoma (CTO)–monocyte (CTG) hybrids (white arrows) from different melanoma cell lines adopt the morphology of monocytes. White bars indicate 10 µm.

**Figure 5:**
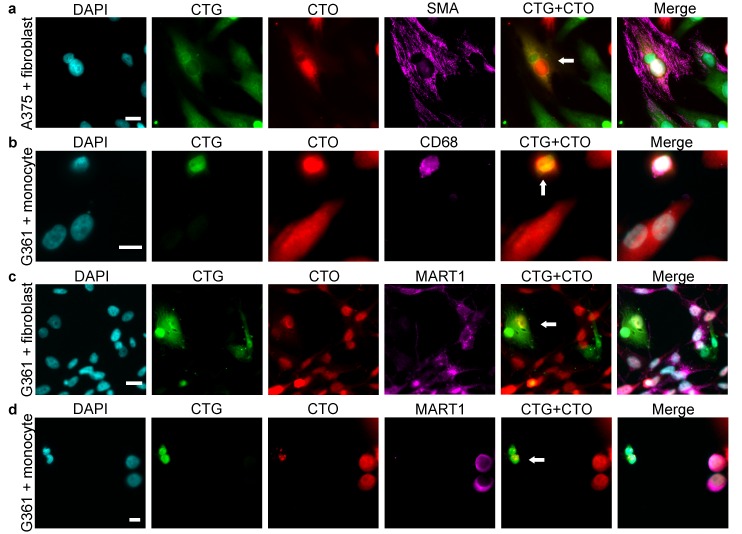
Melanoma–fibroblast and melanoma–monocyte hybrids are indistinguishable from stromal cells based on immunophenotype. (**a**) A melanoma (CTO)–fibroblast (CTG) hybrid cell expressing the fibroblast marker smooth muscle actin (SMA). (**b**) A melanoma (CTO)–monocyte (CTG) hybrid expressing the monocyte-macrophage marker CD68. (**c**,**d**) A melanoma (CTO)–fibroblast (CTG) hybrid (**c**) and a melanoma (CTO)–monocyte (CTG) hybrid (**d**), which do not express the MART1 melanoma marker. White arrows indicate hybrid cells. White bars indicate 20 µm in the case of fibroblasts (**a**,**c**) and 10 µm in the case of monocytes (**b**,**d**).

In all melanoma cell lines used for co-cultures, most of the hybrid cells were indistinguishable from fibroblasts or monocytes based merely on cell size and morphology (Figure 3 and Figure 4). Moreover, all hybrid cells contained one or two nuclei, but no multinucleated cells were detected. Since hybrid cells could adopt the morphology of stromal cells and, more importantly, lose the characteristic morphology of melanoma cells, we examined commonly used phenotypic markers to better characterize the CTG + /CTO + hybrid cells and to investigate whether these markers enable their discrimination from conventional stromal cells. Some melanoma–fibroblast hybrids expressed smooth muscle actin (SMA) (Figure 5a). Similarly, some melanoma–monocyte hybrids were positive for CD68 (Figure 5b). Furthermore, when the MART1-expressing G361 melanoma cell line was used, we detected hybrid cells that lost the expression of MART1 (Figure 5c,d). These results provide evidence that spontaneously formed melanoma–stromal cell hybrids can adopt the morphology and phenotype of stromal cells.

## 3. Discussion

In this paper, we show that human melanoma cells fuse with primary human fibroblasts and human peripheral blood-derived monocytes *in vitro*. By combining cytosolic intravital dyes and *in situ* hybridization techniques, we were able to identify and track the spontaneously formed hybrid cells between melanoma cells and primary stromal cells. This allows the studying of individual spontaneously formed hybrid cells, which is especially interesting, as there are currently no unique sets of markers to identify fused cells *in vivo*. There are only few reports on identifying fused cells in patient’s biopsies [17,18]. These studies pre-select potential hybrid tumor cells using immunohistological approaches and confirm cell fusion events using genetic analyses. Therefore, further *in vitro* work is needed to further characterize and identify markers unique to hybrid cells that would allow their identification *in vivo* as well.

We demonstrate that the fusion takes place spontaneously with fusion rates of more than 0.1%, which are surprisingly high compared to fusion rates reported in previous studies using electro- or chemical fusion, followed by antibiotic selection. One possible explanation for the high fusion rates observed with flow cytometry is that most of the hybrid cells die shortly after formation [8], partly due to mitotic stress, and they may be very sensitive to any additional stress such as antibiotics or sorting. It should be noted that fusion rates in our hands varied significantly among different cell donors and even cell densities. Supposing that hybrid cells are formed constantly at a similar rate *in vivo* in an inflamed tumor microenvironment, even if the majority of the hybrid cells cannot survive, this rate should be sufficient for the detection of hybrid clones *in vivo*.

We found that resulting hybrid cells can adopt the morphology and immunophenotype of stromal cells. Our results are in agreement with other studies proposing that tumor–stromal cell fusion can lead to the formation of hybrid cells displaying mesenchymal traits [6,19]. These findings highlight the fact that hybrid cells could acquire a stromal morphology and phenotype, which is especially interesting, as evidence for the presence of cell fusion in human cancer is from the detection of hybrid cells in the cancer but not the stromal compartments [17,18,20]. Therefore, future investigations are needed to determine whether cancer cell-stromal cell hybrids could be detected in the peritumoral stromal compartments as well. However, the role of spontaneously formed hybrid cells *in vivo* is unknown and needs further investigation.

Several *in vitro* and animal studies suggest that cell fusion might contribute to tumor progression by promoting metastasis formation or by inducing drug resistance [14,21] and that tumor cells can acquire stem-cell-like properties by fusing with stromal cells [6,19,22]. Therefore, cell fusion might serve as an evolutionary mechanism for tumor cells to acquire properties that allow them to react quickly to a changing environment, such as during chemotherapy or irradiation, or even to evade immune recognition. The malignant behavior of these tumor–stromal hybrid cells in mice is supported by the finding that, even though fusion hybrids of tumor cells and multipotent stromal cells first show a predominantly mesenchymal morphology, they subsequently undergo a reversal to a cancer-like phenotype and that these hybrid cells form invasive and metastatic tumors in mice [11]. However, data suggest that most hybrid cells die shortly after formation and even those cells that later start to proliferate stay in a resting state for weeks, which supports the idea of cell fusion being an anti-tumor mechanism, which in some rare cases may give rise to clones displaying even more malignant features than the parental tumor cells [8]. Furthermore, as our results suggest, cell fusion might provide an alternative mechanism for phenotype switching; thus, melanoma cells could become undetectable with standard histopathological methods that rely on the thorough examination of cell morphology and the expression of melanocytic markers.

In conclusion, we have here provided evidence that melanoma cells can adopt the phenotype of stromal fibroblasts and macrophages by spontaneous cell fusion *in vitro*. However, further studies are needed to functionally characterize the hybrid cells, to identify similar cells *in vivo*, and to clarify the role of tumor-stromal cell fusion in tumor progression.

## 4. Experimental Section

### 4.1. Cell Culture

The human melanoma cell lines A375, G361, UACC-257, and SK-MEL-2 were kindly provided by Krisztina Buzás. All cell lines were authenticated using short tandem repeat (STR) analysis (Microsynth AG, Balgach, Switzerland) after finishing all experiments. Primary human monocytes (donated by Zsuzsanna Kurgyis and Lajos V. Kemény) were obtained from peripheral blood and isolated using MagCellect Human CD14^+^ Cell Isolation Kit (R&D Systems, Minneapolis, MN, USA) following gradient centrifugation with Ficoll-Paque (GE Healthcare Life Sciences, Chalfont St Giles, UK). Human dermal fibroblasts were obtained from healthy individuals undergoing plastic surgery with the approval of the local institutional review board and after signing an informed consent. Skin samples were incubated in dispase solution overnight at 4 °C. After the removal of the epidermis, the dermis was incubated in a digestion medium (10.8 mg collagenase, 5 mg hyaluronidase, 0.4 mg deoxyribonuclease, and 0.1 mL fetal bovine serum diluted in 4 mL DMEM low glucose medium) for two hours at 37 °C and filtered through a 100-µm cell strainer.

Each melanoma cell line stained with 6 µmol/mL CellTracker Orange (CMTMR) was mixed either with fibroblasts or with freshly isolated monocytes labeled with 10 and 4 µmol/mL CellTracker Green (CMFDA, both from Life Technologies, Carlsbad, CA, USA), respectively, and grown in co-cultures for 24 h. The fibroblast-containing co-cultures were grown in low glucose DMEM supplemented with 5% fetal bovine serum, while the monocyte-containing co-cultures in RPMI 1640 (HEPES modification) with 10% fetal bovine serum and 30 IU/mL GMCSF (R&D Systems). All growth media were supplemented with 1% Penicillin/Streptomycin and 1% Glutamine. All cell culture reagents used are products of Lonza (Basel, Switzerland), if not specified otherwise.

For confocal microscopic visualization 100,000 melanoma cells were seeded either with 200,000 fibroblasts or with 300,000 monocytes on 1-well Lab-Tek^®^ II Chamber Slides™ (Sigma-Aldrich, St. Louis, MI, USA); for flow cytometric analyses, 20,000 melanoma cells either with 40,000 fibroblasts or with 60,000 monocytes were seeded onto 12-well plates (Corning, Corning, NY, USA).

### 4.2. Detection of Spontaneous Cell Fusion

Co-cultures fixed with 4% paraformaldehyde (PFA) were visualized with Fluoview 1000 (Olympus, Tokyo, Japan) confocal laser-scanning microscope with a 40× oil immersion objective. Excitation/emission wavelengths were 405/461, 488/519, and 543/567 nm for 4′,6-diamidin-2-phenylindol (DAPI), CTG, and CTO, respectively. For *Z* stacking, step size was set to 1 µm. All fluorescent image analyses and three-dimensional reconstructions were performed with ImageJ software (National Insitutes of Health, Bethesda, MD, USA).

### 4.3. Fluorescent in Situ Hybridization

Hybrid cells in PFA-fixed co-cultures were identified using AxioImager.Z1 epifluorescent microscope, AxioCamMR3 camera (both from Carl Zeiss Microscopy, Jena, Germany) and 40× oil immersion objective. Excitation/emission filters were set to 353/465, 495/519, and 558/575 nm for DAPI, CTG, and CTO, respectively. After the removal of cover slips, samples were fixed again with 3:1 ratio of methanol and acetate acid followed by photobleaching using Suntest CPS+ Tabletop Xenon Exposure Systems (Thermo Fischer Scientific, Waltham, MA, USA). The monocyte-containing co-cultures were digested using 0.01N HCl, 0.005% pepsin, and fixed with PFA. All samples were incubated in 70% formamide in 2× SSC at 95 °C, then in 70%, 80%, and absolute alcohol each and dried. The X and Yc dual label fluorescent probes (Reagens Ltd., Pécs, Hungary) were put on the samples, denatured at 72 °C, and incubated overnight at 37 °C. Then, samples were washed with 0.3% NP-40 in 2× SSC and incubated in 0.3% NP-40 in 0.4× SSC at 72 °C. FISH signals were visualized with Fluoview 1000 confocal laser-scanning microscope. Excitation/emission wavelengths were 488/519 and 543/567 nm for X and Y chromosomes, respectively.

### 4.4. Fluorescent Live Cell Imaging

Co-cultures grown for 12 h were put into the chamber of a Fluoview 10i-W confocal laser-scanning microscope (Olympus). Excitation/emission wavelengths were 473/519 and 559/567 nm for CTG and CTO, respectively. Five *Z*-stack images with 3-µm step sizes were taken every 60 min for 12 h at 90 randomly chosen regions using 60× magnification.

### 4.5. Measurements of Cell Fusion Rates

Co-cultures fixed with PFA and stained with DAPI were analyzed in a CyFlow^®^ Space flow cytometer (Sysmex Partec GmbH, Görlitz, Germany). DAPI, CellTracker Green, and CellTracker Orange were detected on channels FL10, FL1, and FL12, respectively. Gating is shown in Figure S3. Assuming that double positive cells in a co-culture result not only from cell fusion, we used transwell cultures for controls, in which monocytes or fibroblasts were separated from melanoma cells by a Transwell^®^ (Corning) with a pore size of 3.0 µm; thus, small, stained dead cell particles could cross the membrane but intact cells could not fuse. Therefore, fusion rate was determined as the rate of double positive cells detected in transwell cultures subtracted from that of co-cultures. All data were analyzed using FlowJo software (FlowJo, Ashland, OR, USA).

### 4.6. Immunofluorescent Staining

Co-cultures were fixed with PFA. High (for MART1) or low (for CD68) pH target retrieval solution (DAKO, Glostrup, Denmark) was used for heat-induced epitope retrieval; for SMA staining, 0.02% Triton-X was used as penetration enhancer. 50% heat-inactivated serum of the monocyte donor was used to block Fc receptors on monocytes and 1% goat serum to prevent the nonspecific binding of the secondary antibody. MART1 (clone 103, 1:200), SMA (clone 1A4, 1:200), and CD68 (clone PG-M1, 1:100) mouse monoclonal antibodies (all from DAKO) and their appropriate isotype controls: mouse anti-human IgG1, IgG2a, and IgG3 (all from Biolegend, San Diego, CA, USA) were labeled with goat anti-mouse Alexa 647 antibody (Life Technologies, 1:400). All the reagents were diluted in tris-buffered saline containing 1% bovine serum albumin and, for monocyte-containing co-cultures, 50% heat-inactivated serum of the monocyte donor.

Immunofluorescent stainings were visualized with epifluorescent microscopy. Excitation/emission filters were set to 653/668 nm for Alexa 647.

### 4.7. Statistical Analysis

One-way analysis of variance and Bonferroni *post hoc* tests were used to compare the means of fusion rates in different melanoma cell lines.

## Supplementary Materials

Supplementary materials can be found at http://www.mdpi.com/1422-0067/17/6/826/s1.

## Abbreviations

The following abbreviations are used in this manuscript:

CTG	CellTracker Green
CTO	CellTracker Orange
MART1	Melanoma antigen recognized by T-cells
SMA	Smooth muscle actin

## References

[1] Harkness T., Weaver B.A., Alexander C.M., Ogle B.M. (2013). Cell fusion in tumor development: Accelerated genetic evolution. Crit. Rev. Oncog..

[2] Clawson G.A. (2013). Cancer. Fusion for moving. Science.

[3] Lu X., Kang Y. (2009). Cell fusion as a hidden force in tumor progression. Cancer Res..

[4] Bjerkvig R., Tysnes B.B., Aboody K.S., Najbauer J., Terzis A.J.A. (2005). Opinion: The origin of the cancer stem cell: Current controversies and new insights. Nat. Rev. Cancer.

[5] Duelli D., Lazebnik Y. (2003). Cell fusion: A hidden enemy?. Cancer Cell.

[6] Xu M.-H., Gao X., Luo D., Zhou X.-D., Xiong W., Liu G.-X. (2014). EMT and acquisition of stem cell-like properties are involved in spontaneous formation of tumorigenic hybrids between lung cancer and bone marrow-derived mesenchymal stem cells. PLoS ONE.

[7] Chakraborty A.K., Sodi S., Rachkovsky M., Kolesnikova N., Platt J.T., Bolognia J.L., Pawelek J.M. (2000). A spontaneous murine melanoma lung metastasis comprised of host × tumor hybrids. Cancer Res..

[8] Wang R., Sun X., Wang C.Y., Hu P., Chu C.-Y., Liu S., Zhau H.E., Chung L.W.K. (2012). Spontaneous cancer-stromal cell fusion as a mechanism of prostate cancer androgen-independent progression. PLoS ONE.

[9] Dittmar T., Schwitalla S., Seidel J., Haverkampf S., Reith G., Meyer-Staeckling S., Brandt B.H., Niggemann B., Zänker K.S. (2011). Characterization of hybrid cells derived from spontaneous fusion events between breast epithelial cells exhibiting stem-like characteristics and breast cancer cells. Clin. Exp. Metastasis.

[10] Martin-Padura I., Marighetti P., Gregato G., Agliano A., Malazzi O., Mancuso P., Pruneri G., Viale A., Bertolini F. (2012). Spontaneous cell fusion of acute leukemia cells and macrophages observed in cells with leukemic potential. Neoplasia.

[11] Rappa G., Mercapide J., Lorico A. (2012). Spontaneous formation of tumorigenic hybrids between breast cancer and multipotent stromal cells is a source of tumor heterogeneity. Am. J. Pathol..

[12] Lu X., Kang Y. (2009). Efficient acquisition of dual metastasis organotropism to bone and lung through stable spontaneous fusion between MDA-MB-231 variants. Proc. Natl. Acad. Sci. USA.

[13] Özel C., Seidel J., Meyer-Staeckling S., Brandt B.H., Niggemann B., Zänker K.S., Dittmar T. (2012). Hybrid cells derived from breast epithelial cell/breast cancer cell fusion events show a differential RAF-AKT crosstalk. Cell Commun. Signal..

[14] Nagler C., Hardt C., Zänker K.S., Dittmar T. (2011). Co-cultivation of murine BMDCs with 67NR mouse mammary carcinoma cells give rise to highly drug resistant cells. Cancer Cell Int..

[15] Helming L., Gordon S. (2009). Molecular mediators of macrophage fusion. Trends Cell Biol..

[16] Terada N., Hamazaki T., Oka M., Hoki M., Mastalerz D.M., Nakano Y., Meyer E.M., Morel L., Petersen B.E., Scott E.W. (2002). Bone marrow cells adopt the phenotype of other cells by spontaneous cell fusion. Nature.

[17] Lazova R., LaBerge G.S., Duvall E., Spoelstra N., Klump V., Sznol M., Cooper D., Spritz R.A., Chang J.T., Pawelek J.M. (2013). A melanoma brain metastasis with a donor-patient hybrid genome following bone marrow transplantation: First evidence for fusion in human cancer. PLoS ONE.

[18] Yilmaz Y., Lazova R., Qumsiyeh M., Cooper D., Pawelek J. (2005). Donor Y chromosome in renal carcinoma cells of a female BMT recipient: Visualization of putative BMT-tumor hybrids by FISH. Bone Marrow Transplant..

[19] Ding J., Jin W., Chen C., Shao Z., Wu J. (2012). Tumor associated macrophage × cancer cell hybrids may acquire cancer stem cell properties in breast cancer. PLoS ONE.

[20] Shabo I., Midtbö K., Andersson H., Åkerlund E., Olsson H., Wegman P., Gunnarsson C., Lindström A. (2015). Macrophage traits in cancer cells are induced by macrophage-cancer cell fusion and cannot be explained by cellular interaction. BMC Cancer.

[21] Rachkovsky M., Sodi S., Chakraborty A., Avissar Y., Bolognia J., McNiff J.M., Platt J., Bermudes D., Pawelek J. (1998). Melanoma × macrophage hybrids with enhanced metastatic potential. Clin. Exp. Metastasis.

[22] Clawson G.A., Matters G.L., Xin P., Imamura-Kawasawa Y., Du Z., Thiboutot D.M., Helm K.F., Neves R.I., Abraham T. (2015). Macrophage-tumor cell fusions from peripheral blood of melanoma patients. PLoS ONE.

