# Supplementary material for: Melanoma Cells Can Adopt the Phenotype of Stromal Fibroblasts and Macrophages by Spontaneous Cell Fusion in Vitro
Source: Int J Mol Sci. 2016 Jun 2;17(6):826. doi: 10.3390/ijms17060826 (PMC4926360; doi:10.3390/ijms17060826)
Supplement: Supplementary file 1 [file ijms-17-00826-s001.zip › ijms-127528-Supplementary Materials/ijms-127528-supplementary-publish.pdf]

Supplementary Materials: Melanoma Cells Can Adopt the Phenotype of Stromal Fibroblasts and Macrophages by Spontaneous Cell Fusion *in Vitro*

Lajos V. Kemény, Zsuzsanna Kurgyis, Tünde Buknicz, Gergely Groma, Ádám Jakab, Kurt Zänker, Thomas Dittmar, Lajos Kemény and István B. Németh

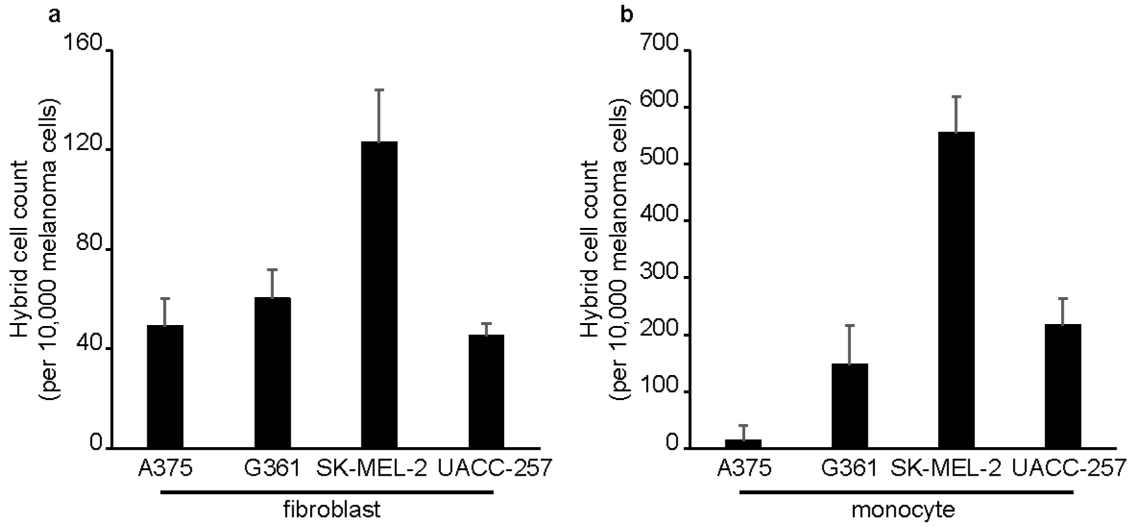

Figure S1. Hybrid cell counts in different melanoma cell lines. Melanoma–fibroblast (**a**) and melanoma–monocyte (**b**) hybrid cell counts compared to 10,000 melanoma cells after 24 h of co-culture measured with flow cytometry. Means of three experiments + SD are shown.

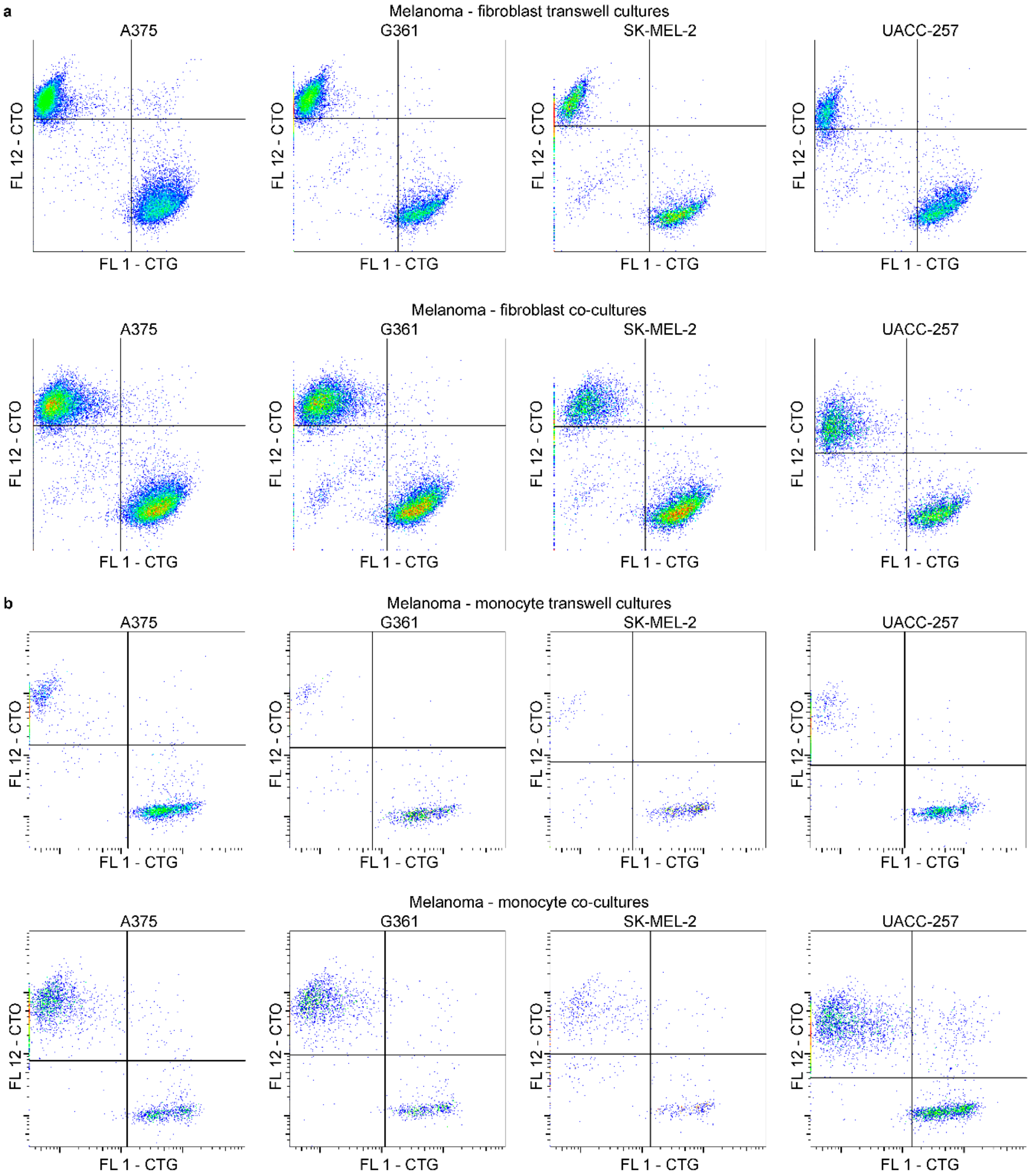

Figure S2. All investigated melanoma cell lines fuse spontaneously with human dermal fibroblasts and peripheral blood derived monocytes. (**a**,**b**) Representative pseudocoloured dot plots of melanoma-fibroblast (**a**) and melanoma-monocyte (**b**) transwell (**upper rows**) and co-cultures (**lower rows**) from three experiments. Since the different cells could only fuse in co-cultures but not in transwell cultures, fusion rate was determined as the rate of double positive cells detected in transwell cultures subtracted from that of co-cultures.

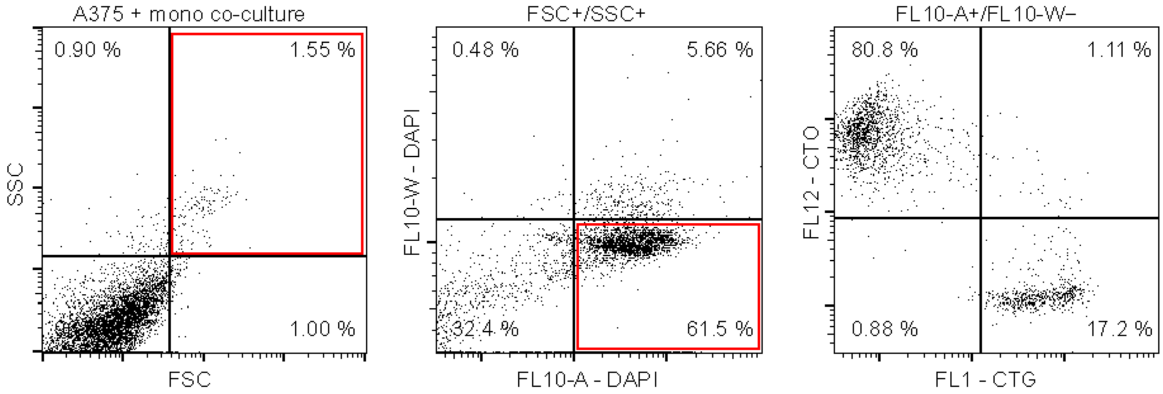

Figure S3. Gating mechanisms in flow cytometry for the measurement of cell fusion rates. Representative dot plots of an A375-monocyte co-culture. In the forward scatter (FSC)–side scatter (SSC) plot (**left panel**) cell debris is excluded upon gating the events in the upper right quadrant. In the FL10-A–FL10-W plot (**middle panel**) only the FSC + /SSC + events are displayed. FL10-A − cells are considered as apoptotic cells while FL10-W + cells as cell doublets and are therefore excluded. Only FL10-A + /FL10-W − cells are displayed in the CellTracker Green (CTG)–CellTracker Orange (CTO) plot (**right panel**), in which CTG + /CTO − cells are monocytes and CTG − /CTO + cells are melanoma cells.
